# Childhood Pneumonia Screener: a concept

**DOI:** 10.1007/BF03371463

**Published:** 2015-12-01

**Authors:** Keith Grimwood

**Affiliations:** Grifth University and Gold Coast University Hospital, Gold Coast, QLD Australia

**To the Editor:**

I congratulate Räsänen and Gavriely for their recent thought-provoking article describing how mobile ‘smart’ phone technology could assist in diagnosing and managing pneumonia in children from developing countries lacking well-established healthcare systems and infrastructure [1]. In the article they describe their plans for first developing, then validating, implementing and finally evaluating a pneumonia screening tool, which if successful could act as a model for improving healthcare delivery in resource-poor settings. Many pneumonia-related deaths in children are avoidable by not only addressing malnutrition, promoting breastfeeding, improving household standards and immunisation, but also from prompt recognition, providing antibiotics when necessary, correcting hypoxaemia, fluid and nutritional deficits, treating any co-morbidities and monitoring for complications. The proposed pneumonia screening tool mobile phone application could therefore help support primary healthcare workers to better diagnose and manage childhood pneumonia in rural regions of developing countries where both health infrastructure and training are limited. Such an achievement would benefit global child health since pneumonia in developing countries is the most important cause of mortality and morbidity in young children and when recurrent, it is also linked to future chronic lung disease [2].

I would be interested though in learning more about the planned large-scale field evaluation of the pneumonia screening tool. Do the authors, for example intend to conduct a cluster-randomised controlled trial? This methodology has been adopted previously to determine the feasibility and efficacy of health service type interventions in sub-Saharan Africa, such as the mass distribution of azithromycin to help control trachoma [3]. The authors state the primary outcome of this trial will be the impact upon mortality. However, this has been difficult to measure in intervention studies for pneumonia, especially comparative antibiotic treatment trials, because of study design where few participants have had bacterial pneumonia [4] and from being conducted in settings where 60–80% of deaths occur outside hospital [5,6]. These factors should be taken into consideration when designing their study. Indeed, it is hoped the pneumonia screening tool will help to better identify children with pneumonia requiring antibiotics and/or supportive care, while with time mobile network coverage should increase in rural areas of Africa and Asia providing those most vulnerable with access to this technology. Nevertheless, it will be important to include additional outcome measures, such as antibiotic courses, transfer to another healthcare facility, alternative diagnoses and cost-benefit analyses as integral components of any large, population-based evaluation trial.

Finally, as limited healthcare facilities and failure to recognise severe illness are important reasons for childhood deaths in developing countries [7], specially designed ‘smart’ phone applications could assist parents and caregivers with identifying ‘danger signals’ in young children and knowing when to seek urgent medical attention. Such devices will rely upon extended cellular network coverage in remote regions, improved population literacy, the capacity to recharge phones and minimising consumer costs. Nevertheless, the outlook is promising and, for example, in Nigeria 90% of the population can access mobile networks, even though one-third still lack electricity and one-fifth are without clean water [8].

**Competing interests:** The author declares no competing interests.

## References

[1] Räsänen J, Gavriely N (2014). Childhood pneumonia screener: a concept. pneumonia.

[2] Chang AB, Ooi MH, Perera D, Grimwood K (2013). Improving the diagnosis, management, and outcomes of children with pneumonia: where are the gaps?. Front Pediatr.

[3] Porco TC, Gebre T, Ayele B, House J, Keenan J, Zhou Z (2009). Effect of mass distribution of azithromycin for trachoma control on overall mortality in Ethiopian children: a randomized trial. JAMA.

[4] Mulholland K, Carlin JB, Duke T, Weber M (2014). The challenges of trials of antibiotics for pneumonia in low-income countries. Lancet Respir Med.

[5] Nair H, Simões EA, Rudan I, Gessner BD, Azziz-Baumgartner E, Zhang JS (2013). Severe Acute Lower Respiratory Infections Working Group. Global and regional burden of hospital admissions for severe acute lower respiratory infections in young children in 2010: a systematic analysis. Lancet.

[6] Berkley JA, Lowe BS, Mwangi I, Williams T, Bauni E, Mwarumba S (2005). Bacteremia among children admitted to a rural hospital in Kenya. N Engl J Med.

[7] Niederman MS, Krilov LR (2013). Acute lower respiratory infections in developing countries. Lancet.

[8] Intelligence GS (2014). The mobile economy.

